# What drives adult vaccine hesitancy? Content and face validation of a new measure for U. S. Adults

**DOI:** 10.3389/fpubh.2026.1819321

**Published:** 2026-08-06

**Authors:** Divya S. Subramaniam, Michael P. Poirier, Elizabeth Ward, Mintesnot T. Teni, Amanda L. Meiklejohn, Dipti P. Subramaniam

**Affiliations:** 1 Department of Health and Clinical Outcomes Research, Saint Louis University School of Medicine, St. Louis, MO, United States; 2 Duo Scientia, LLC, St. Louis, MO, United States; 3 Department of Epidemiology, George Washington University Milken Institute School of Public Health, Washington, DC, United States; 4 Advanced HEAlth Data (AHEAD) Institute, Saint Louis University School of Medicine, St. Louis, MO, United States; 5 Gateway Business Health Coalition, St. Louis, MO, United States; 6 College for Public Health and Social Justice, Saint Louis University, St. Louis, MO, United States

**Keywords:** content validity, face validity, health outcomes measurement, instrument validation, population health, vaccine hesitancy

## Abstract

**Background:**

Vaccine hesitancy is viewed as a complex interaction of behavioral and societal factors that can intervene in the uptake of vaccines or lack thereof. To date, no existing instrument developed for the U. S. adult population measures general vaccine hesitancy in a way that integrates behavioral and cognitive determinants and generalizes across vaccines rather than targeting a specific disease. The development of this tool will be useful for clinicians to counsel patients on vaccination and for vaccine related public health interventions targeted at improving population health.

**Methods:**

Based on a comprehensive review of the literature, 38 items were developed to be rated on a 5-point Likert scale. Eight subject matter experts across the United States completed a content validity review to determine the relevance of survey items. Face validity feedback was collected from three self-reported vaccine-hesitant, one with a neutral stance on vaccines, and three not vaccine-hesitant individuals.

**Results:**

Item-Level CVI (I-CVI) ranged from 0.38 to 1.00 for the initial 38 items. Five items were dropped due to low CVI scores, face validity feedback, and similarity to other items. Four items were revised to incorporate the viewpoints of comparable items, address face validity feedback, and improve overall clarity and alignment with the survey’s objectives. Lastly, two items below the I-CVI threshold of 0.80 were retained after the research team deemed them necessary for addressing general vaccine hesitancy in the adult population. The Scale-Level CVI (S-CVI) was 0.87 across all 38 items initially rated and 0.90 for the final 33-item scale, indicating strong content validity, and the final survey consists of 33 items.

**Conclusion:**

This tool lays the groundwork for better understanding the barriers associated with vaccination and hesitancies that prevent the growth of herd immunity and overall vaccination uptake. Pilot testing on a large sample of the adult population is required to refine the tool items further.

## Introduction

Vaccine hesitancy is defined as the refusal or delay of vaccination uptake despite available services to be vaccinated (1–5). Vaccination demonstrates effectiveness in preventing the contraction of communicable diseases (6). It is estimated that every year, adequate vaccination saves six lives every minute (7). Despite these positive health outcomes associated with vaccination, data from the 2022 National Health Interview Survey indicate that only 22.8% of adults aged ≥19 years had received all age-appropriate vaccines included in a composite adult vaccination measure (8).

Vaccine hesitancy is often discussed as though it were a single phenomenon, but the reasons people give for delaying or declining vaccination differ in kind, not merely in degree. A person who believes vaccines cause the diseases they are meant to prevent is responding to misinformation about biological mechanisms (9). A person who cannot take time off work to reach a vaccination site is facing a logistical barrier unrelated to belief (10). A person who distrusts vaccination because of documented mistreatment of their community by the healthcare system is responding to a historical and systemic reality rather than a misconception (11, 12). These are distinct problems that happen to produce the same observable outcome, and a measurement approach that does not distinguish among them risks treating separate public health challenges as though they were interchangeable.

An abundance of research on vaccine hesitancy has been conducted, which focused on vaccination hesitancy toward pneumococcal, influenza, human papillomavirus (HPV), dengue, and SARS-CoV-2 vaccines (5, 13–15). The World Health Organization (WHO) identified suboptimal vaccination coverage despite fully funded or accessible vaccine services as a public health issue in 2019 and named it one of the top 10 threats to global health (16). Reasons for refusing vaccination vary widely, depending on the individual and their experiences. As such, vaccine hesitancy impedes progress toward ongoing disease prevention and creates negative consequences for public health (17, 18).

These consequences are best understood at the population level, where the effects of individual vaccination decisions accumulate. Vaccines provide acquired immunity, acting as preventative measures against contracting infectious diseases. When a substantial portion of the population is vaccinated, the likelihood of individuals contracting infectious diseases decreases, known as herd immunity. This limits the number of people requiring hospital beds and other medical interventions (19). However, decreased rates of vaccine uptake have contributed to widespread outbreaks of severe preventable diseases (20). Vaccine hesitancy is one of the leading reasons for decreased uptake of vaccination (21).

Researchers have investigated a range of complex societal and individual factors, and trends that influence vaccine hesitancy and uptake globally. Micro (e.g., attitudes, physician recommendations, families, and peers) and meso-level factors (e.g., neighborhood/environment, institution policies, and practices) were analyzed to show that enhancing positive beliefs and perceptions can have a favorable impact on vaccination uptake (10). The Vaccine Hesitancy Scale (VHS) was developed by the World Health Organization Strategic Advisory Group of Experts on Immunization (WHO SAGE) (22). Adaptations of the VHS have been developed to address a wide range of disease and population-specific vaccinations. For example, the adult Vaccine Hesitancy Scale (aVHS) was developed from the VHS, relative to influenza vaccine uptake and COVID-19 vaccination acceptance (17). Adaptations of the VHS show reliability for evaluating rates of vaccine hesitancy among the adult population specifically for COVID-19 and general parental attitudes toward vaccination (18, 23). Despite the existence of downstream applications of the VHS, to our knowledge, there is a lack of developed theoretical frameworks that gage vaccine hesitancy among the general adult population in a way that is applicable to various diseases (24).

Given the diversity of the adult population, vaccine hesitancy experiences also vary. Therefore, it is essential to understand and analyze all factors that may influence their perceptions. These associated factors can include race, education, annual income, ethnicity, age, gender, sexual orientation, religious and political affiliations, and geographic location (25). Furthermore, by understanding the varying levels of trust in vaccinations, health and clinical officials can enhance the understanding of psychosocial predictors such as knowledge and social norms (26, 27). Previous research has attempted to understand these perceptions but struggled to fully isolate responses that incorporate parental viewpoints, healthcare workers’ perspectives, and how perceptions of specific vaccinations can be generalized to understand overall vaccine hesitancy among adults (28, 29).

Existing studies have focused on healthcare workers’ opinions and perceptions of vaccinations, especially during peak influenza seasons but may be biased due to their occupation (30). Knowledge and healthcare experiences can positively skew data and inaccurately represent the overall adult population. Additionally, research focusing on specific vaccinations may present hesitancies and perceptions that are specific to that disease and vaccination. For example, human papillomavirus (HPV) vaccinations more commonly garner dialog from parents, as the CDC states that HPV vaccination is recommended for children between 11 and 12 years old (31). By assessing key drivers, such as perceived susceptibility and benefits of getting vaccinated, different vaccinations can prompt unconventional responses that cannot be universalized.

While vaccine and population-specific findings can be useful, our study addresses a notable gap in the literature by focusing primarily on the often-neglected viewpoint and perceptions of the general adult population toward general vaccine hesitancy. Existing VHS-derived instruments were designed primarily to quantify hesitancy toward a particular vaccine or within a specific population and to estimate its prevalence, rather than to characterize, within a single general-purpose measure, the behavioral and cognitive determinants that drive hesitancy across vaccines. This leaves a distinct measurement gap that a general, theoretically grounded instrument is positioned to address. Therefore, this study seeks to develop a novel tool to assess general adult vaccine hesitancy, independent of confounding influences that may otherwise obscure underlying attitudes. Through this approach, this research aims to systematically examine the multifaceted determinants shaping vaccine related perceptions and decision-making drivers among adults in the United States. A single instrument that captures hesitancy across different vaccines and subpopulations, rather than one calibrated to a specific disease, is what makes it possible to compare the drivers of hesitancy on a common scale and to detect shifts in attitude that disease-specific measures would otherwise miss.

## Materials and methods

### Guiding theoretical frameworks

Existing tools measuring vaccine hesitancy have been developed using a variety of theoretical and conceptual frameworks. Among these, the Health Belief Model (HBM) is the most widely utilized, particularly for understanding motivations behind health-related behaviors. The HBM posits that health behavior decisions are shaped by four core constructs: perceived susceptibility (one’s belief about personal risk of disease), perceived severity (beliefs about the seriousness of disease), perceived benefits (beliefs about the effectiveness of a health action in reducing risk), and perceived barriers (beliefs about the costs or impediments to that action), which are commonly applied to examine vaccination behaviors (32). Another prominent framework is the 3C Model, developed by the SAGE Vaccine Hesitancy Working Group of the World Health Organization. This model identifies three key factors: complacency (low perceived risk of vaccine-preventable disease, leading to low prioritization of vaccination), confidence (trust in vaccine safety, efficacy, and the health systems delivering vaccines), and convenience (the degree to which physical, financial, and information access to vaccines affects uptake), that serve as primary contributors to vaccine hesitancy and behavior (33, 34).

While these two frameworks address complementary dimensions of vaccine hesitancy, neither alone is sufficient for comprehensive measurement. The 3C model captures population-level behavioral drivers but does not systematically address the individual cognitive appraisals of disease risk and vaccination benefits that are central to the HBM. Conversely, the HBM provides a comprehensive account of individual-level risk cognition but does not explicitly address structural access factors or system-level trust. This study integrates constructs from both frameworks within a reflective measurement model, in which each domain is conceptualized *a priori* as a latent construct manifested by its corresponding items. This proposed structure is treated as a hypothesis to be tested empirically in the validation phases that follow rather than as an established property of the tool.

### Phase one: item generation and refinement process

The concept of general vaccine hesitancy in the U. S. adult population was explored through a thorough literature review and examination of existing survey tools used to gage perceptions and hesitancies for a variety of vaccines. The three domains derived from the 3C Model of Vaccine Hesitancy, confidence, complacency, and convenience, directly informed the first three item domains, with each domain’s items operationalizing the corresponding 3C construct (34). A fourth domain, Vaccination Risk–Benefit Perception, was added to incorporate HBM constructs, perceived susceptibility, perceived severity, perceived benefits, and perceived barriers, that are theoretically distinct from the 3C domains and necessary for a comprehensive account of vaccine hesitancy (30, 35). This domain also includes items related to trust in health communication sources and beliefs about community health, which emerged from the literature as important attitudinal dimensions not covered by the 3Cs (17, 18, 36–38). Table 1 presents the explicit mapping of theoretical constructs to domains and representative items. The four-point scale described in Phase Two below was used only by SMEs to rate item relevance for content validity. In future pilot testing, the final 33-item tool will be administered to respondents using a five-point Likert scale, consistent with the format reported in the Abstract.

**Table 1:** General adult vaccine hesitancy perception domains and items.

	Confidence Items
Item #	Item
1	I take recommendations from healthcare professionals.
2	I trust my personal doctor’s recommendations regarding vaccinations.
3	Vaccines are safe.
4	Vaccines are effective at preventing disease.
5	Vaccines have harmful side effects.
6	I refused a vaccine recommended by my physician because I considered it dangerous.
7	I am hesitant to receive vaccinations due to harmful side effects.
8	I have experienced side effects after vaccination.
9	The benefits of vaccination outweigh the risks.
10	I am knowledgeable about vaccines.
11	I have information on when to get vaccinated, who should get vaccinated, and harmful side effects.
12	Vaccines cause the diseases against which they protect.
13	Vaccines trigger diseases like autism, multiple sclerosis, and diabetes.
14	I have trust in official healthcare authorities’ (e.g., the Centers for Disease Control and Prevention, and the U. S. Food and Drug Administration) guidelines on healthcare issues.
15	I think the government is equipped to provide proper vaccination recommendations.
16	I trust pharmaceutical companies to make safe and effective vaccines.
27	Individuals in my racial group have had negative experiences with vaccination.
Convenience Items	
Item #	Item
17	I have access to vaccinations.
18	I have time to get vaccinated.
19	I know that vaccinations are accessible to me.
20	I know locations in my community where I can get vaccinated.
Complacency Items	
Item #	Item
21	I am against vaccination.
22	Vaccination can prevent the contraction of serious infectious disease.
23	Vaccines are beneficial for my overall health.
24	Newly developed vaccines carry more risk than older vaccines.
25	Vaccines weaken the immune system.
26	I trust the science and research related to vaccination.
27	Individuals in my racial group have had negative experiences with vaccination.
28	I use alternative medicine instead of vaccines to protect against diseases.
29	If there is a risk for disease, I am inclined to get vaccinated.
30	I know someone who has gotten sick from a vaccine-preventable disease.
31	I receive annual vaccinations (e.g., influenza).
32	Vaccines should be mandatory in employment settings.
33	Vaccines should be mandatory in school settings.
34	Vaccines should never be mandatory.
Vaccine Risk-Benefit Perception Items	
Item #	Item
35	Vaccines preserve the health of my community.
36	The information I receive about vaccines from healthcare organizations (e.g., the Centers for Disease Control and Prevention, and the U. S. Food and Drug Administration) is reliable.
37	I do not need vaccines for diseases that are not common anymore.
38	I do not need vaccines for diseases that are not severe anymore.

The initial review and assessment of the literature yielded 297 items across the four domains. Following this step, the number of items was then narrowed down to 69 items after repeated and comparable questions were removed. Several items were created by consolidating multiple items into a singular item concerning the shared overarching perception. The third round of review was conducted to ensure each item assessed the correct perception, leaving 52 remaining items to be reviewed. Individual item ratings (i.e., keep, rephrase, or discard) were given by the authors and resulted in 46 items. A second round of individual item ratings was conducted by the entire research team following the initial rating. The final number of items is 38 for the content validity assessment. Each stage of this reduction required deliberate judgment rather than mechanical elimination: items were removed when two statements appeared to measure the same underlying belief from different angles, and items were retained when they captured a facet of hesitancy, such as distrust rooted in prior experience versus unfamiliarity with a newly developed vaccine, that no other item addressed. This iterative winnowing is the substantive work of instrument development, since a tool can only detect meaningful differences in hesitancy to the extent that this kind of judgment was exercised in deciding what counts as a conceptually distinct item in the first place (39).

### Phase two: content validity assessment

To assess the content validity of the general vaccination hesitancy tool, phase two involved the construction and dissemination of a content validity survey to subject matter experts (SMEs). SMEs were selected based on demonstrated scholarly or applied expertise in vaccine development, vaccine hesitancy, and vaccination behavior and decision-making, with current affiliation at accredited academic or research institutions. Experts were recruited from across the United States to ensure representation of diverse institutional affiliations, disciplinary backgrounds, and areas of specialization. Demographic data was not collected, as eligibility was determined based on professional background and subject matter expertise. This eight-member panel falls within the range of five to 20 experts commonly recommended for content validity assessment and reflects the emphasis in the methodological literature on selecting panelists with demonstrated scholarly or applied standing in the content area (40–43). This study engaged subject matter experts exclusively for content validity evaluation and did not constitute human subjects research. The experts and lay reviewers who took part provided methodological feedback to refine the instrument rather than serving as subjects of study. Institutional Review Board approval was not required because the activity focused solely on obtaining feedback regarding the clarity, relevance, and usability of the instrument rather than collecting research data on the study topic. No identifiable or sensitive participant data were collected or analyzed. Content and face validity assessments are generally considered part of instrument development and not human subjects research requiring IRB oversight (39, 44).

A survey of the initial items was created via Qualtrics® and utilized a 4-point Likert scale of 1 = *not relevant*, 2 = *somewhat relevant*, 3 = *quite relevant*, and 4 = *highly relevant*. Eight SMEs completed the content validity survey via Qualtrics®. The CVI was calculated for the relevancy of 38 items. The Item-Level CVI (I-CVI) was calculated for each survey item, and the Scale-Level CVI (S-CVI) was calculated for the overall survey tool. In addition to rating each item in the tool, SMEs could leave feedback or comments on each survey item and overall feedback after the content validity survey. The formulas used to calculate I-CVI and S-CVI scores are as follows (45):

- I-CVI = N of experts rating items a 3 or 4 / Total N of experts
- S-CVI (Average) = Final average of all I-CVI scores

Because this phase evaluated item relevance through independent expert ratings rather than coder agreement on a shared qualitative dataset, inter-rater reliability statistics such as Cohen’s or Fleiss’ kappa were not applicable; I-CVI and S-CVI are the standard and sufficient metrics for evaluating content validity at this stage of instrument development (39, 40, 46). Feedback from the SMEs led to confirmation or removal of items from the survey tool (Table 1). This content validity survey presented the complete list of items, a crucial step to inform the final survey tool and validate earlier decisions in determining domains for survey items (i.e., confidence, convenience, complacency, and vaccine risk–benefit perception).

### Phase three: utilization of face validity

To further validate the tool, phase three focuses on whether the tool genuinely reflects the experiences and insights of the general adult population. Face validity ensures participant understanding of tool items and identifies any confusing, ambiguous, or irrelevant themes before rigorous testing. This approach helps to ensure that the population being studied is accurately represented (47). This study was limited to the assessment of face validity by lay individuals who were professional contacts of the research team. Participants were not recruited as research subjects, no intervention was conducted, and no identifiable private or confidential information was collected. As the activity did not constitute human subjects research, IRB review and approval were not required (39, 44).

Face validity assessment relied on a convenience-based sample (*n* = 7), and several methodological limitations should be acknowledged. Participants were known to the research team, which may have introduced selection bias and increased the risk of social desirability bias in their feedback. These limitations are inherent to the preliminary nature of this phase and are explicitly recognized. The use of individuals known to the research team was intentional and aligned with common practices in early-stage instrument development. Recruiting trusted contacts facilitated rapid, iterative feedback on item clarity, wording, and interpretability in a low-burden setting, prior to more resource-intensive pilot testing. Importantly, participants were informed that their feedback (positive or critical) was essential for improving the instrument, and responses were collected without identifiers to help reduce potential response bias. This approach is consistent with ethical research practices for minimal-risk activities focused on tool refinement, where no sensitive personal data is collected. This number is consistent with previous research, knowing that larger samples can show diminishing returns in identifying major problems with the survey items (39, 48, 49). A total of seven participants gave feedback on the items, three identified as vaccine-hesitant, one identified as neutral stance on vaccines, and three identified as not vaccine-hesitant. Vaccine hesitancy status was determined through self-identification. Participants were asked to indicate whether they considered themselves hesitant about vaccines; no formal or validated scale was applied at this stage. This approach was used to ensure inclusion of perspectives from individuals who perceive themselves as hesitant, which is particularly relevant for assessing the clarity and acceptability of items. The face validity assessment focused on participants’ understanding of item content, wording, and relevance. No formal cognitive interviewing techniques were employed, as participants were simply asked to provide written answers to the following questions: (1) Do these statements and questions regarding vaccine hesitancy make sense? (2) Did we miss any hesitancy or perception items that are important to consider? and (3) Do you have any other thoughts?

## Results

Thirty-eight items were developed through a comprehensive literature review and were evaluated for content validity and face validity. Thirteen SMEs in vaccine hesitancy, perception, and uptake were invited to participate in the CVI assessment, with eight completing it, resulting in a 62% response rate. Feedback was collected anonymously to ensure unbiased responses, and all participants met predefined inclusion criteria. I-CVI scores ranged from 0.38 to 1.00, with a threshold of 0.80 indicating strong item validity (46). Most items achieved scores of 0.88 or higher, exceeding the threshold for excellent content validity (39, 40, 46). Items scoring below 0.80 were reviewed and either revised, retained, or excluded. An overall Scale-Level CVI Average (S-CVI Average) of ≥0.90 is considered excellent (39, 40, 46). For our survey tool, the S-CVI across all 38 items initially rated was 0.87, indicating strong content validity, and the S-CVI computed across only the 33 retained items reached 0.90, meeting the 0.90 threshold these sources classify as excellent (39, 40, 46).

Qualitative feedback from face validity reviewers was examined in conjunction with CVI results to guide item refinement and consolidation of the final survey tool. Responses regarding clarity were positive overall, and readers stated that most potential determinants of general adult vaccine hesitancy were thoroughly represented. Selected examples of feedback are provided below for illustration:

- “*I think a blanket statement toward the beginning such as ‘I consider myself pro/anti-vaccine’ may help”*.
- “*Some of your questions are similar but capture slightly different nuances*”.

As outlined in Table 2, twenty-nine items scored an I-CVI ≥ 0.80. Twenty-seven items were deemed excellent due to high I-CVI scores and are included in the final survey tool (39, 40, 46). Item 5 (harmful side effects) received a score of 1.00, but it was removed after face-validity feedback suggested it was too similar to Item 7 (hesitancy and harmful side effects). Item 7 received a 1.00 and was revised for clarity based on face validity feedback (despite having excellent content validity). Seven items received an I-CVI score of 0.75. Of those 7 items, 2 were retained, 3 were revised, and 2 were removed. Items 21 (against vaccination) and 34 (should never be mandatory) were retained despite I-CVI < 0.80, after careful consideration by the authors, who determined that each item captured a distinct construct not otherwise represented in the item set: Item 21 assesses outright vaccine opposition, and Item 34 assesses categorical rejection of vaccine mandates, neither of which is addressed by any other retained item. These two items are retained provisionally to preserve content coverage of outright vaccine opposition and categorical rejection of mandates; both are flagged for re-evaluation, and possible removal, during psychometric testing, where their empirical performance will determine whether they remain in the final scale. Items 12 (causal associations), 17 (access to vaccination), and 23 (beneficial for overall health) all scored 0.75 and were revised for improved clarity and alignment with the survey’s objectives. Items 32 (mandatory for employment) and 33 (mandatory for schools) both scored 0.75 and were removed based on face validity feedback stating the items were redundant given other items surrounding vaccine mandates. Items 10 (personal knowledge) and 18 (time to get vaccinated) received an I-CVI score of < 0.75, scoring below our inclusion threshold, and were excluded from the survey. Following this iterative process of evaluation and refinement, the final general adult vaccine hesitancy tool comprises 33 items.

**Table 2:** Content validity index for items of general adult vaccine hesitancy and perception tool.

Item #	No. of SMEs agreeing on relevance (rated 3 or 4)	I-CVI	CVI interpretation	Item revision
1	7	0.88	Excellent	
2	8	1.00	Excellent	
3	8	1.00	Excellent	
4	8	1.00	Excellent	
5	8	1.00	Removed	
6	7	0.88	Excellent	
7	8	1.00	Excellent*	I am afraid of harmful side effects because of receiving a vaccination.
8	7	0.88	Excellent	
9	7	0.88	Excellent	
10	3	0.38	Removed	
11	7	0.88	Excellent	
12	6	0.75	Revised	Vaccines cause the disease they are designed to protect against.
13	8	1.00	Excellent	
14	7	0.88	Excellent	
15	7	0.88	Excellent	
16	8	1.00	Excellent	
17	6	0.75	Revised	I have the means (e.g., money and time) to receive vaccinations.
18	5	0.63	Removed	
19	7	0.88	Excellent	
20	8	1.00	Excellent	
21	6	0.75	Retained	
22	8	1.00	Excellent	
23	6	0.75	Revised	Vaccines improve my overall health.
24	7	0.88	Excellent	
25	7	0.88	Excellent	
26	8	1.00	Excellent	
27	7	0.88	Excellent	
28	7	0.88	Excellent	
29	7	0.88	Excellent	
30	7	0.88	Excellent	
31	8	1.00	Excellent	
32	6	0.75	Removed	
33	6	0.75	Removed	
34	6	0.75	Retained	
35	8	1.00	Excellent	
36	7	0.88	Excellent	
37	7	0.88	Excellent	
38	7	0.88	Excellent	

*Item revised based on face validity feedback despite having excellent content validity value.

## Discussion

The item set produced through this development process illustrates directly why vaccine hesitancy resists treatment as a single construct. An item asking whether respondents believe vaccines “cause the diseases they are designed to protect against” captures susceptibility to biological misinformation (9). An item asking whether respondents “have the means (e.g., money and time) to receive vaccinations” captures a logistical barrier with no bearing on belief or trust (10). An item asking whether “individuals in my racial group have had negative experiences with vaccination” captures a historical and systemic form of distrust that is not a misconception to be corrected but a documented reality to be addressed (11, 12). Each of these items can plausibly contribute to the same behavioral outcome, non-vaccination, yet each implies a different explanation and, by extension, a different public health response: correcting a false biological claim, removing an access barrier, and rebuilding institutional trust are not interchangeable interventions. This heterogeneity is the central reason a general-purpose measurement tool has value: it allows researchers and practitioners to identify which of these mechanisms is operating in a given population, rather than treating hesitancy as a uniform attitude to be persuaded away.

This heterogeneity also plays out against a broader public health backdrop. Vaccine hesitancy poses a significant global public health challenge, undermining vaccination uptake and disease prevention efforts across diverse populations (16–18, 21). The drivers of hesitancy are multifaceted, encompassing individual psychological factors such as anxiety and coping, societal influences, and healthcare system dynamics, all of which interact with sociodemographic characteristics to shape vaccination decisions (10, 18, 25, 50–54). Addressing these concerns requires tailored approaches, such as listening to individual fears, building trust, and adapting public health messaging to evolving attitudes and circumstances (55). Specific populations, such as cancer survivors, older adults, and individuals with chronic conditions, present unique challenges that influence their vaccination decisions (50, 53, 54). Healthcare providers, who are often trusted sources of information, play a critical role in addressing hesitancy and encouraging acceptance (56). These approaches are increasingly grounded in established frameworks, such as the Health Belief Model (HBM) and the 3C model of confidence, convenience, and complacency, which together capture the breadth of psychological and contextual factors influencing vaccine decision-making (57).

A reliable tool to measure general vaccine hesitancy among adults could be valuable for understanding and addressing this issue more holistically. Vaccine hesitancy arises from a complex mix of psychological, social, cultural, and systemic influences, and a well-designed instrument may capture these dynamics in ways that support public health surveillance and research. Such a tool could potentially enable identification of trends in vaccine hesitancy across populations and over time, thereby informing public health resource allocation (22, 58, 59). At the clinical level, understanding patient-level drivers of hesitancy such as fears about vaccine safety could support tailored communication strategies, pending further validation of the instrument’s clinical utility (60, 61). At the health system level, aggregated data might describe population attitudes and beliefs and inform community-based outreach and culturally responsive interventions (62). From a policy perspective, a validated instrument could eventually be incorporated into evaluation protocols for policy-supported interventions (63). Monitoring sociodemographic disparities in vaccine attitudes may also support equity-focused strategies (64, 65), and a standardized tool could facilitate longitudinal research evaluating scalable public health interventions (66, 67).

This study addresses a gap in the literature by developing a content valid tool to measure general vaccine hesitancy among U. S. adults through a rigorous, multi-phase item development process. Several rounds of item review by the research team and feedback from subject matter experts (SMEs) ensured that items were relevant and comprehensive. Input from both vaccine-hesitant and vaccinated individuals enhanced the tool’s clarity and face validity. The iterative refinement process resulted in a content-valid tool with a strong Scale-Level Content Validity Index (S-CVI) of 0.87, indicating strong expert agreement on item relevance and representativeness (39, 40, 46). Establishing content validity in this staged way, before any psychometric testing, is not a preliminary formality but a determinant of what the finished instrument can credibly measure, since an item that is ambiguous, redundant, or conceptually misplaced cannot be recovered through later statistical modeling. It must be noted, however, that content validity alone does not establish construct validity, reliability, or clinical utility; these properties require empirical evaluation in subsequent psychometric study phases.

A key methodological consideration in the development of this tool concerns the conceptual demarcation between overlapping theoretical constructs, particularly between items grounded in HBM dimensions such as perceived susceptibility, severity, benefits, and barriers and those capturing broader attitudinal perceptions of vaccines. While these domains are theoretically distinct, their operationalization in survey items can produce conceptual and empirical overlap, which may affect the tool’s factorial structure. For example, an individual’s perceived risk of vaccine side effects may simultaneously reflect HBM constructs of perceived barriers and broader distrust perceptions, making clean separation across domains challenging. Items addressing trust in government and manufacturers, attitudes toward vaccine mandates, and group-based experiences with the healthcare system are retained because they map onto well-established determinants of hesitancy in the SAGE framework, in which confidence encompasses trust in the vaccine, in the system that delivers it, and in the motivations of policymakers, and in which contextual influences encompass historical and sociopolitical experience. These items therefore capture recognized drivers of hesitancy rather than extraneous political attitudes. A concrete instance of this challenge arose during item classification itself: Item 27 (“Individuals in my racial group have had negative experiences with vaccination”) was originally grouped under Complacency, but on review was reassigned to Confidence, since it reflects historical and systemic distrust of the vaccination system rather than a low perceived risk of disease (11, 12). This reassignment reflects a broader point about the construct being measured: hesitancy rooted in a community’s prior mistreatment by the healthcare system is psychologically distinct from hesitancy rooted in low perceived urgency or logistical inconvenience, even though a single quantitative response to a survey item cannot always distinguish between them. Item-level decisions like this one are therefore not a peripheral bookkeeping exercise but a substantive part of instrument development, since misclassifying an item can obscure exactly the kind of etiological distinction that a general population hesitancy tool is meant to capture. At this stage of development, a formal measurement model has not yet been specified or tested. The dimensional structure of the tool, whether it is best represented as a unidimensional scale or a multidimensional instrument with correlated subscales, remains an empirical question to be addressed through exploratory and confirmatory factor analyses in the next phase of future validation studies. Investigators should therefore be cautious about interpreting domain scores as distinct constructs until the tool’s structural validity is established.

The strengths of this study include the extensive review of existing literature, the grounding of items in established theoretical frameworks, and the use of a rigorous content validation process involving both expert review and cognitive input from members of the target population. These methodological steps support the content validity of the tool as a foundational step in a multi phase instrument development process. However, several important limitations must be acknowledged. First, this tool was deliberately developed for use within the U. S. adult population, and its item content, language, and underlying assumptions reflect the cultural, institutional, and healthcare context of the United States. As such, the tool is intended, in its current form, exclusively for use with U. S. adult populations. Generalizability to other national or cultural contexts cannot be assumed, and the tool should not be applied internationally without appropriate adaptation. Should future research seek to extend the use of this tool to other countries or languages, rigorous cross-cultural adaptation procedures would be required prior to implementation. These would include forward-backward translation, bilingual expert panel review, cognitive interviewing with members of the target population, and full psychometric re-validation to ensure conceptual, linguistic, and measurement equivalence across diverse cultural and healthcare settings (39, 68, 69).

Second, the demographic composition of participants involved in the item development, content and face validity phases was not systematically reported in this study, which limits the ability to evaluate whether diverse racial, ethnic, socioeconomic, educational, and geographic perspectives within the U. S. were adequately represented. Future validation studies must recruit demographically, and geographically diverse samples drawn from across the United States to ensure the tool’s content and structural validity are not limited to a narrow population subgroup. Third, and critically, the clinical utility, policy relevance, and population health applications discussed in this paper are speculative at this stage. Without empirical validation including assessment of internal consistency, test–retest reliability, construct validity, and sensitivity to change, the tool’s performance in real-world clinical or public health settings cannot be assumed. Claims regarding its ability to guide clinical communication, community outreach, or policy evaluation should be interpreted as aspirational goals for future research rather than demonstrated capabilities of the current instrument.

Building on this foundation, the next phase of this research will include pilot testing followed by comprehensive psychometric evaluation in a larger, demographically and geographically diverse U. S. sample to establish reliability, validity, and generalizability within the intended population. The pilot study will assess construct validity through exploratory factor analysis, evaluate item clarity and feasibility, and examine preliminary item performance, informing any necessary refinements prior to full scale validation. This pilot will also incorporate formal cognitive interviewing with a larger, independent, and demographically diverse U. S. sample to systematically evaluate how respondents interpret each item, strengthening response-process validity beyond the preliminary face-validity input obtained during item development. In the larger validation study, internal consistency and test–retest reliability will be evaluated to ensure temporal stability, and confirmatory factor analysis will be conducted to test the tool’s dimensional structure and determine whether the theoretically proposed domains are empirically supported. These analyses will also assess the discriminant validity of items addressing institutional trust, vaccine mandates, and group-based experiences by examining their associations with established measures of institutional trust and sociopolitical attitudes, testing whether these items operate as determinants of vaccine hesitancy rather than as indicators of a separate construct. Importantly, demographic diversity within the U. S. validation sample will be systematically documented and reported to enable assessment of generalizability across population subgroups. Should the tool demonstrate strong psychometric properties within the U. S., future research may explore cross-cultural adaptation for use in other countries and languages.

Collectively, these next steps will strengthen the evidentiary foundation of the adult vaccine hesitancy tool and clarify the timeline for any eventual clinical, surveillance, or policy application. In conclusion, this study represents a foundational contribution to the measurement of adult vaccine hesitancy in the United States. Grounded in established theoretical frameworks and developed through a rigorous multi-phase content validation process, it achieves an S-CVI of 0.87 and advances a more nuanced understanding of hesitancy as a construct rooted in trust, perception, lived experience, and systemic barriers rather than a simple knowledge gap. In its current form, the tool should be regarded as a content valid instrument at the item development stage. Its clinical utility, policy applicability, and broader public health value will be established through the psychometric validation phases that follow. If those phases confirm the instrument’s reliability, structural validity, and construct validity, it holds meaningful potential as a resource for hesitancy surveillance, tailored intervention design, and evidence informed public health and policy decision making across diverse U. S. populations and, ultimately, beyond.

## Data Availability

The original contributions presented in the study are included in the article/supplementary material, further inquiries can be directed to the corresponding author/s.
